# *In vitro* polyploid induction in* Allium grayi* Regel using colchicine

**DOI:** 10.7717/peerj.20790

**Published:** 2026-02-12

**Authors:** Ting-Syuan Chen, Wei-Hung Zhang, Si-Yu Liu, Cheng-Ying Hung, Chien-Yuan Kao

**Affiliations:** 1 Department of Horticulture, National Ilan University, Yilan, Taiwan; 2 Hualian District Agricultural Research and Extension Station, Ministry of Agriculture, Hualian, Taiwan

**Keywords:** Plant tissue culture, Tetraploid, Chimras, Bulb, Shallot

## Abstract

*Allium grayi* Regel is a seasonally limited small perennial herb of the subfamily *Allioideae*, and is endemic to Matsu, Taiwan. The species possesses nutritious, ornamental value, and biological pharmacological activity. This study evaluated for the first time to induce polyploidy using the dissected bulb of *A. grayi* as explants *in vitro*, with the expectation of increasing the bulb size. Sterile bulbs were divided into four equal parts and pre-cultured under different durations before being soaked in a one g/L colchicine solution for 12 or 24 hours. Survival, regeneration, variation, and tetraploid induction rates were recorded, while ploidy levels were determined by flow cytometry and stomatal traits were measured microscopically. The results showed that pre-cultured treatment after dissecting the sterile bulbs increased the variation rate of the plants, with the group that was left static for 10 days before immersing in a one g/L colchicine liquid medium for 24 hours showing the best results. The variation rate reached 100%, and the induction rate of tetraploid plants reached 20% by flow cytometry examination. Among 123 regenerated shoots, 13 were chimeras and three stable tetraploid lines were established, all exhibiting larger stomata and lower stomatal density than diploids. These tetraploid lines provide great potential for future breeding and improvement of Matsu native shallot cultivars.

## Introduction

**Figure 1:**
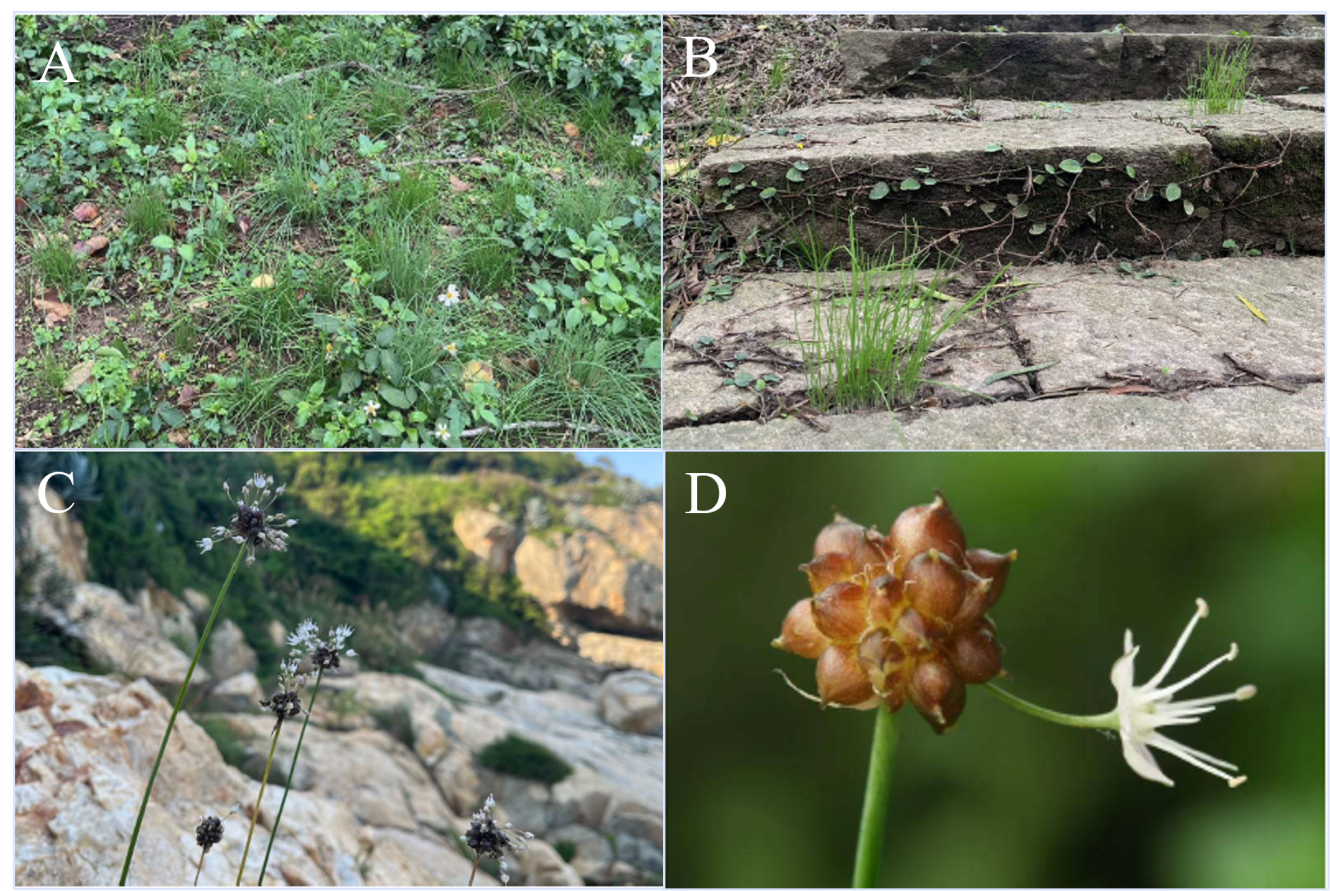
Growth conditions of wild shallot in Matsu, Taiwan. (A) Many shallot plants can be found in open areas without tree shade; (B) on stone steps that are not walked on; and (C) in rock crevices, indicating their high drought resistance. (D) The inflorescences often bear a large number of bulbils. Photo credit: Lienchiang county Farmer’s Association.

The *Amaryllidaceae* family consists of perennial flowering monocots, encompassing 73 genera and over 1,000 species, primarily distributed in tropical and subtropical regions. The *Amaryllidaceae* family is further divided into three subfamilies: *Agapanthoideae*, *Allioideae*, and *Amaryllidoideae*. Several major genera in *Amaryllidaceae* family include *Agapanthus*, *Allium*, *Clivia*, and *Narcissus*, all of which possess ornamental, economic value, and biological pharmacological activity (Meerow, 2023). Members of the *Allium* genus exhibit diverse biological activities. In particular, *Allium grayi* Regel (Fig. 1)—also referred to as *Allium macrostemon* in some taxonomic treatments—has been reported to possess antioxidant, antibacterial, lipid-lowering, anti-alopecia, antidepressant, and anti-obesity effects (Lee et al., 2010; Gao et al., 2023; Kim, Lee & Kim, 2023; Li et al., 2023). These findings highlight the pharmacological potential of this species complex and support its traditional and contemporary use in both medicinal and culinary applications.

Polyploidy refers to the presence of chromosome sets in a cell nucleus that exceed the diploid level (Ren et al., 2018). Polyploid plants exhibit larger morphological traits, fewer stomata, and increased secondary metabolites compared to diploid plants. Additionally, polyploid plants can overcome hybridization barriers and enhance resistance to stress and disease. Colchicine was originally extracted from the plant Colchicum. In the first century AD, Pedanius Dioscorides described colchicine extracts as a treatment for gout in his work “De Materia Medica”, and it is now commonly used to prevent gout attacks (Kundu & Ray, 2017). However, colchicine is also a chemical mutagen used for inducing polyploidy; its mechanism involves binding to tubulin, which prevents spindle fibers from pulling sister chromatids to opposite poles of the cell, leading to DNA replication without cell division and resulting in chromosome doubling (Hailu et al., 2021).

Previous studies have demonstrated successful colchicine-induced tetraploidy in several Allium species, including garlic (*A. sativum*), leek (*A. ampeloprasum*), using root tips, shoot meristems, stem discs, or callus tissues as explants (Adaniya & Tamaki, 1991; Kafawin & Chen, 1991; Hailu, Wiendi & Dinarti, 2020). However, the use of bulb scales as explants for *in vitro* polyploid induction has been rarely reported. Furthermore, the physiological status of explants—particularly the influence of pre-culture or inoculation duration prior to colchicine treatment—has not been systematically examined in *Allium*. To date, no studies have addressed this approach in *Allium grayi* or closely related shallot species.

In a study of garlic originating from Indonesia, “Tawangmangu Baru”, soaking stem disc explants in 0.1% colchicine for 24 and 48 h resulted in the successful production of tetraploid plants (Hailu, Wiendi & Dinarti, 2020). Similarly, colchicine treatment of *A. cepa* L. var. aggregatum G. Don explants resulted in changes in ploidy level and plant morphology (Ren et al., 2018). In other bulbous species, pre-culture treatments have been shown to play a critical role in polyploid induction efficiency (Li et al., 2020; Kafawin & Chen, 1991). These findings suggest that a period of pre-culture may enhance the physiological readiness of explant tissues, thereby improving the efficiency of chromosome doubling. However, no published reports exist on the *in vitro* propagation and polyploid induction of the *A. grayi*. The present study therefore represents the first attempt to evaluate the effects of pre-culture duration on colchicine-induced polyploidy in *Allium grayi*, aiming to optimize induction efficiency and provide a basis for its future breeding and germplasm improvement.

This study reports the first successful *in vitro* induction of polyploidy on Matsu native shallot, with particular emphasis on increasing the pre-culture period of fresh dissected bulbs prior to colchicine treatment. We specifically tested whether prolonged pre-culture enhances the induction efficiency and cytological stability of tetraploids and whether tetraploid plants exhibit morphological and stomatal features consistent with genome duplication. The outcomes of this work establish a methodological foundation for efficient polyploid induction in *A. grayi*, contributing to germplasm enhancement, conservation, and the future development of larger, high-value bulbs with enriched bioactive compounds.

## Materials & Methods

### Plant materials

The bulbs of the native Matsu shallot used in this study were collected by staff from the Lienchiang County Farmers’ Association and served as experimental materials. About 50 healthy bulbs showing no signs of injury or disease were selected and used as explant sources for subsequent *in vitro* culture. First, the roots of the bulbs were removed to create a flat base. The bulbs were then rinsed with tap water and soaked in 75% ethanol with shaking for 3 min, followed by soaking in a 10 g/L (1% w/v) NaOCl (CLOROX, USA) aqueous solution with one to two drops of Tween 20 for 15 min with shaking, and finally rinsed in sterile water for 15 min. The bulbs were cultured in full-strength MS (Murashige & Skoog, 1962) medium, which contained 30 g/L sucrose, one mg/L 6-BA, 0.1 mg/L NAA, and three g/L Gelrite. Before autoclaving, the pH was adjusted to 5.7 ± 0.1. The cultures were maintained under a light intensity of 45 µmol m^−^^2^ s^−^^1^, a photoperiod of 10/14 h (light/dark), and a temperature of 24 ± 1 °C.

### Colchicine concentration and soaking time on polyploid induction

The method was modified from Dixit & Chaudhary (2014). Sixteen bulbs were prepared and individually divided into four equal sections, which were then immersed in colchicine solutions at different concentrations (0, 1, 5, and 10 g/L) for 6, 12, 24, and 48 h, respectively. After soaking, the dissected bulbs were rinsed three times with sterile water and then cultured in a shoot induction medium composed of full-strength MS supplemented with 30 g/L sucrose, one mg/L 6-BA, and six g/L Agar. The pH of the medium was adjusted to 5.7 ± 0.1 before autoclaving. Each treatment consisted of four replicates, and each replicate corresponded to one-quarter of a bulb. The cultures were maintained under a light intensity of 45 µmol m^−^^2^ s^−^^1^, a photoperiod of 10/14 h (light/dark), and a temperature of 24 ± 1 °C. After eight weeks, the regenerated shoots were individually subcultured onto a bulblet enlargement medium consisting of full-strength MS supplemented with 60 g/L sucrose, 10 mg/L 6-BA, and six g/L agar. The pH of the medium was adjusted to 5.7 ± 0.1 before autoclaving. Explant survival rate was recorded as the proportion of viable explants producing shoots after culture. After one month of cultivation, the leaves were used for stomatal observation and flow cytometric analysis, and the variation and induction rates were subsequently calculated. The survival rate was defined as the number of explants producing shoots divided by the total number of explants ×100; the variation rate was calculated as (number of mixoploid and tetraploid shoots/total regenerated shoots) ×100; and the induction rate was calculated as (number of tetraploid shoots/total regenerated shoots) ×100.

### Short-term inoculation treatment for polyploid induction

The method was modified from Jeloudar et al. (2019). A total of 10 sterilzed bulbs were each divided into four equal sections and cultured in a shoot induction medium for 4, 8, 12, and 24 h, respectively. After that, the bulbs were soaked in a shoot induction liquid medium containing one g/L colchicine for 12 h. The bulbs were then rinsed three times with sterile water and cultured in a shoot induction medium. Each treatment consisted of eight replicates, and each replicate corresponded to one-quarter of a bulb. The cultures were maintained under a light intensity of 45 µmol m^−^^2^ s^−^^1^, a photoperiod of 10/14 h (light/dark), and a temperature of 24 ± 1 °C. After 30 days, the regenerated shoots were individually subcultured onto a bulblet enlargement medium. Explant survival rate was recorded as the proportion of viable explants producing shoots after culture. After one month of cultivation, the leaves were used for stomatal observation and flow cytometric analysis, and the variation and induction rates were subsequently calculated.

### Long-term inoculation treatment for polyploid induction

A total of 12 sterilized bulbs were each divided into four equal sections and cultured in a shoot induction medium for 1, 3, 5, 7, and 10 days, respectively. After this, the bulb scales were soaked in a shoot induction liquid medium containing one g/L colchicine for 24 h. The bulb scales were then rinsed several times with sterile water and cultured in a shoot induction medium. Each treatment consisted of eight replicates, and each replicate corresponded to one-quarter of a bulb. The cultures were maintained under a light intensity of 45 µmol m^−^^2^ s^−^^1^, a photoperiod of 10/14 h (light/dark), and a temperature of 24 ± 1 °C. After 30 days, the regenerated shoots were individually subcultured onto a bulblet enlargement medium. Explant survival rate was recorded as the proportion of viable explants producing shoots after culture. After one month of cultivation, the leaves were used for stomatal observation and flow cytometric analysis, and the variation and induction rates were subsequently calculated.

### Stomatal calculation

A one cm section of *in vitro* native Matsu shallot leaf was placed on a glass slide and cut open using a scalpel to expose a flat surface. The mesophyll was scraped away, leaving only the epidermal layer. After adding 1–2 drops of deionized water, a coverslip was placed on top, and the sample was observed under a light microscope (40x = 4*10 x). The stomatal calculation involved randomly capturing three images under a microscope, counting the number of stomata in the images, and averaging the results. Stomatal size and density were analyzed using ImageJ software.

### Flow cytometry

Under aseptic conditions, approximately 0.5 cm^2^ leaf segments were excised from each regenerated plant for subsequent flow cytometric analysis. The previously excised leaf segments were placed in a Petri dish, followed by the addition of 250 µL Cystain PI extraction buffer. The plant tissue was minced using a razor blade for 30 to 60 s. Then, one mL of nuclear staining solution (prepared with 20 mL of Galbraith buffer, 120 µL of PI solution, and 60 µL of RNaseA stock solution) was added. The mixture was filtered through a 30 µm filter into a test tube, incubated in the dark at room temperature for 30 min, and analyzed for ploidy level using a flow cytometer (CytoFLEX S, Beckman Coulter, Brea, CA, USA), following the protocol of Doležel, Greilhuber & Suda (2007) with minor modifications.

### Statistical analysis

The data calculations and statistics for the experiments were performed with Microsoft Excel 365 software and the Statistic Analysis System (SAS) software package was applied to Duncan’s multiple range test. The level of significant difference between treatments was defined at 5%.

## Results

### Effects of colchicine concentration and soaking duration on polyploid induction

Colchicine treatment significantly affected the survival and variation rates of Matsu native shallot explants (Table 1). Soaking in one g/L colchicine solution for six hours resulted in a 100% survival rate. However, as the colchicine concentration and soaking duration increased, the survival rate gradually decreased. The survival rate remained at 100% in all control groups (0 g/L colchicine), while in the one g/L treatment group, survival decreased to 75% at 12 and 24 h and dropped to 0% at 48 h. Furthermore, a chimera (mixoploid) was observed in the group soaked in a one g/L colchicine solution for 12 h, while a tetraploid emerged in the group soaked for 24 h, with a variation rate of 20% and an induction rate of 20%. The five g/L and 10 g/L colchicine treatments resulted in even lower survival rates, with 50% survival at 6 h for five g/L, but 0% survival at all longer durations and at all time points for 10 g/L. On the other hand, the group soaked in a one g/L colchicine solution for 6 h had a survival rate of 100%, but the number of regenerated plants was significantly lower than that of the control group. These findings indicate that colchicine concentration and soaking duration interact to influence the survival rate of explants. Furthermore, colchicine treatment has a certain level of toxicity, which negatively affects the plant regeneration ability.

**Table 1:** The effects of different concentrations and treatment durations of colchicine on the regeneration of native Matsu shallot.

Colchicine (g/L)	Time (hr)	No. shoot	Average number of shoots	Chimeric	Tetraploid	Variation rate (%)	Induction rate (%)	Survival rate (%)
0	6	43	10.75 ± 0.96	–	–	–	–	100
	12	42	10.5 ± 1.29	–	–	–	–	100
	24	40	10 ± 1.83	–	–	–	–	100
	48	40	10 ± 2.94	–	–	–	–	100
1	6	13	3.25 ± 1.25	–	–	–	–	100
	12	10	2.5 ± 1.91	1	–	10	–	75
	24	5	1.25 ± 0.96	–	1	20	20	75
	48	–	–	–	–	–	–	0
5	6	3	0.75 ± 0.96	–	–	–	–	50
	12	–	–	–	–	–	–	0
	24	–	–	–	–	–	–	0
	48	–	–	–	–	–	–	0
10	6	–	–	–	–	–	–	0
	12	–	–	–	–	–	–	0
	24	–	–	–	–	–	–	0
	48	–	–	–	–	–	–	0

**Notes.**

*n* = 4, Cultivation for 8 week.

### Effects of short-term inoculation treatment on polyploid induction

Based on the previous experiment, one g/L colchicine was determined to be the optimal concentration for polyploid induction in Matsu native shallot. However, since chimeras were found in the 12-hour soaking group, this experiment pre-cultured quartered shallot bulb scales in shoot-inducing medium for 0, 4, 8, 12, and 24 h before soaking them in one g/L colchicine liquid shoot-inducing medium for 12 h. This design aimed to investigate whether a short inoculation period before colchicine treatment would influence polyploidy induction and improve mutation stability. A total of six experimental groups, including the control, were established. After 30 days of cultivation, results showed no significant effect of pre-culture duration on survival rate (Table 2). The survival rates for all groups varied from 50% to 75%, with the highest survival observed in the 4-hour and 24-hour pre-culture groups (75%) and the lowest in the 12- hour pre-culture group (50%). Additionally, one chimera was observed in the group pre-cultured for four hours, with a variation rate of 7.69%.

**Table 2:** Effects of different short-term static treatments on induction of polyploidy *in vitro* of *Allium grayi*.

Colchicine (g/L)	Resting time (hr)	No. shoot	Average number of shoots	Chimeric	Tetraploid	Variation rate (%)	Induction rate (%)	Survival rate (%)
1	0	14	2.8 ± 0.84	–	–	–	–	62.5
	4	13	2.17 ± 1.47	1	–	7.69	–	75
	8	7	1.4 ± 0.55	–	–	–	–	62.5
	12	4	1 ± 0	–	–	–	–	50
	24	11	1.83 ± 0.75	–	–	–	–	75

**Notes.**

*n* = 8, Cultivation for 30 Days.

### Effects of long-term inoculation treatment on polyploid induction

The previous experiment showed that short-term pre-culture had no significant effect on polyploid induction in Matsu native shallot. Therefore, this experiment extended the pre-culture duration to 1, 3, 5, 7, and 10 days. Additionally, the colchicine soaking duration was increased to 24 h to reduce the number of regenerated shoots while enhancing the mutation rate of explants. A control group with no pre-culture was also included, forming six experimental groups in total. This experiment aimed to determine whether an extended inoculation period could promote stable polyploid induction by allowing explants to recover before colchicine exposure. After 30 days of cultivation, results indicated no significant effect of pre-culture duration on survival rate (Table 3). The survival rate ranged from 12.5% to 62.5%, with the highest survival in the 0-day and 1-day pre-culture groups (62.5%) and the lowest in the 3-day pre-culture group (12.5%). However, as pre-culture duration increased, the mutation rate significantly increased, reaching its highest in the 10-day pre-culture group. This group produced five regenerated shoots, including four chimeras and one tetraploid, resulting in a 100% variation rate (5/5) and a 20% induction rate. Additionally, the 7-day pre-culture group had a variation rate of 66.67%, and the 5-day group had a variation rate of 50%, showing a clear trend of increased mutation frequency with longer pre-culture duration. The results suggest that longer pre-culture durations may provide more stable conditions for polyploid induction, paralleling previous studies on Allium species where colchicine treatment efficiency varied based on tissue preparation methods and exposure duration.

**Table 3:** Effect of different long term resting on polyploid induction of dissected Matsu Shallot.

Colchicine (g/L)	Resting time (Day)	No. shoot	Average number of shoots	Chimeric	Tetraploid	Variation rate (%)	Induction rate (%)	Survival rate (%)
1	0	14	2.8 ± 0.45	1	1	14.29	7.14	62.5
	1	11	2.2 ± 0.45	1	–	9.09	–	62.5
	3	3	3 ± 0	1	–	33.33	–	12.5
	5	6	3 ± 1.41	3	–	50	–	25
	7	3	1.5 ± 0.71	2	–	66.67	–	25
	10	5	1.67 ± 0.58	4	1	100	20	37.5

**Notes.**

*n* = 8, Cultivation for 30 Days.

### Differences between tetraploid and diploid plants

**Figure 2:**
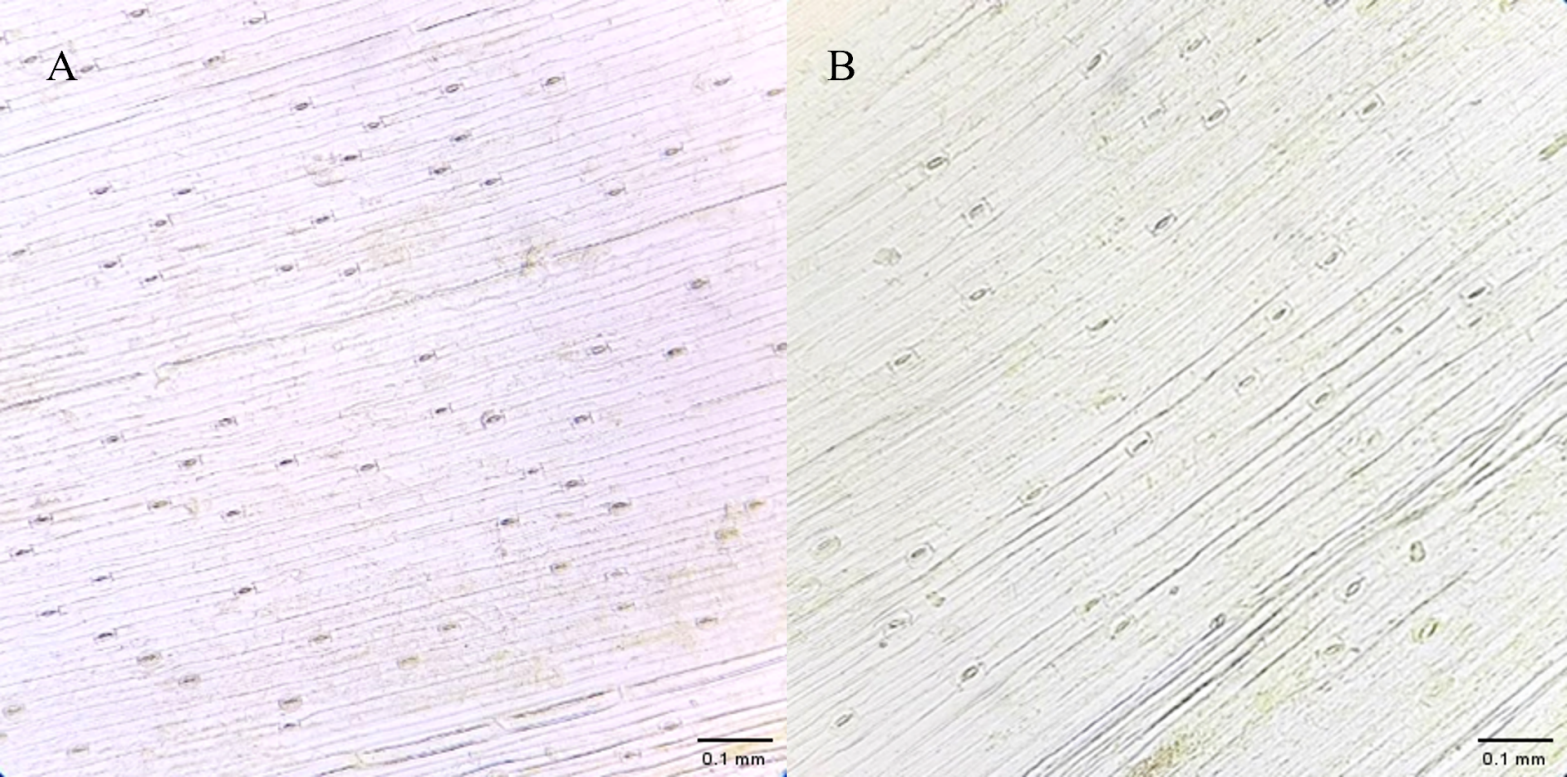
Comparison of stomatal density and size between diploid and tetraploid plants of native Matsu shallot. (A) Diploid plant; (B) tetraploid plant. Bar = 0.1 mm.

**Figure 3:**
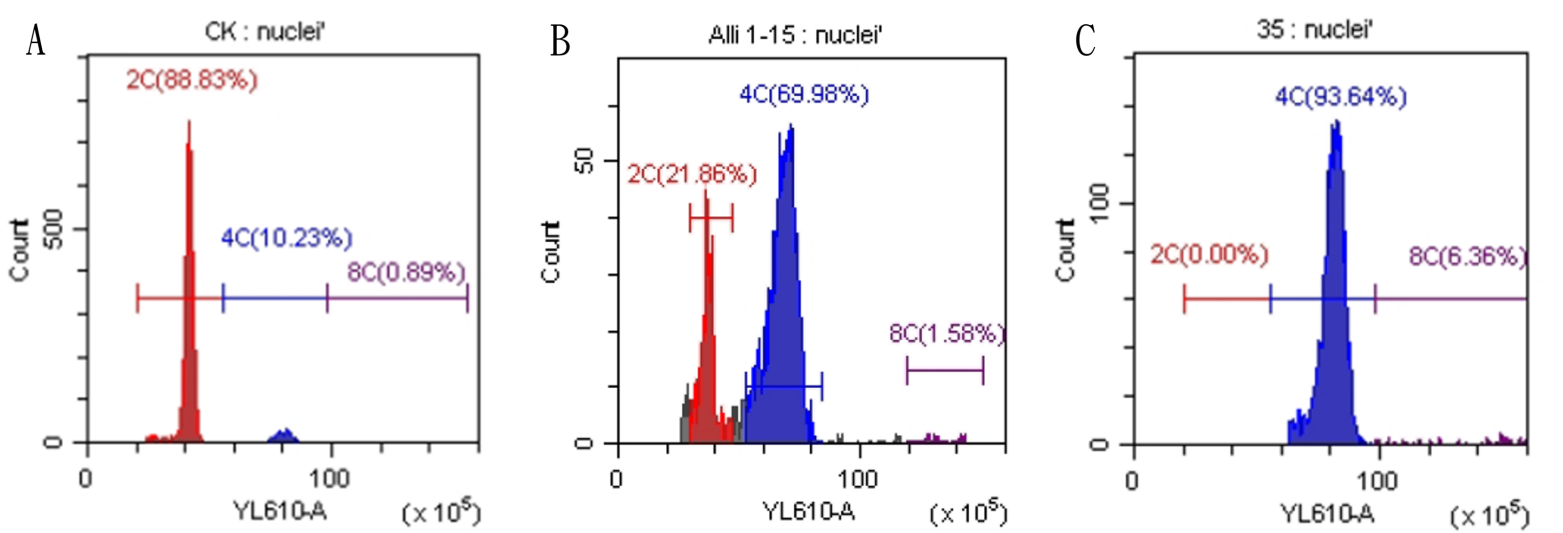
Results of flow cytometry analysis of diploid and tetraploid plants of Matsu native shallot. (A) Diploid plants; (B) chimeric plants; (C) tetraploid plants.

**Figure 4:**
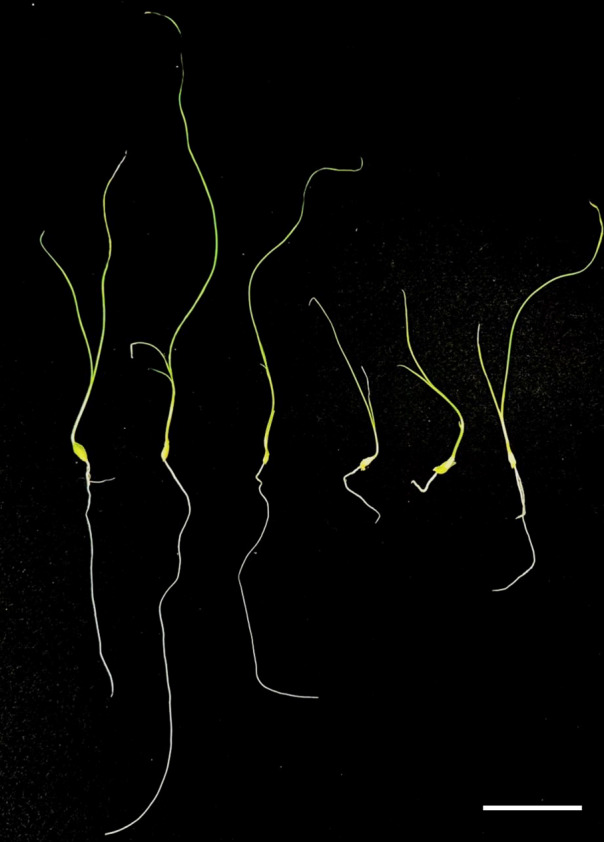
Preliminary comparison between diploid and tetraploid plants after one month of *in vitro* culture. The diploid plant (left) and the tetraploid plant (right) show distinct differences in growth. The tetraploid plant exhibits reduced leaf and root length, indicating a slower growth rate compared to the diploid. Bar = five cm.

Regenerated plants treated with colchicine were analyzed using stomatal characteristics and flow cytometry to confirm the presence of tetraploid plants. Comparisons of stomatal characteristics between tetraploids and diploids (2*n* = 16) showed that the stomatal length of tetraploids was larger, reaching 0.042 mm (Table 4, Fig. 2). Additionally, stomatal density in tetraploids was lower, measuring 14.6431/mm^2^. Flow cytometry confirmed the ploidy levels of the plants, revealing a significant increase in DNA content in tetraploids (Fig. 3). Some regenerated shoots exhibited peaks at both 2c and 4c. If the ratio of 2c to 4c was 3:7 or if the 4c peak was higher, the plant was identified as a chimeric. In addition, diploid and tetraploid plants with bulbs of equal size were cultured *in vitro* for one month to observe differences in early growth performance. Preliminary observations indicated that tetraploid plants exhibited slower growth compared to diploids, as evidenced by shorter leaf and root length (Fig. 4).

**Table 4:** Results of stomatal differences between diploid and tetraploid.

	Number of stomata (count)	Stomatal density (count/mm^2^)	Stomatal length (mm)	Stomatal width (mm)
Diploid	67 a^*^	20.4393 ± 0.9320 a	0.027 ± 0.0040 b	0.010 ± 0.0040 a
Tetraploid	48 b	14.6431 ± 2.3032 b	0.042 ± 0.0017 a	0.012 ± 0.0015 a

**Notes.**

*n* = 3.

* The same letters following values in the same column indicate no significant difference at the 5% level according to Duncan’s multiple range test.

## Discussion

The findings demonstrated a clear dose- and time-dependent response of Matsu shallot explants to colchicine treatment. Low concentrations (one g/L) and shorter exposure times (≤6 h) maintained a high regeneration ability with 100% survival, whereas higher concentrations or longer exposure times drastically reduced viability, consistent with the cytotoxic nature of colchicine. A similar phenomenon was also reported by Kong et al. (2014), who found that an appropriate colchicine concentration and soaking time can stimulate polyploid induction in plants, but higher concentrations and prolonged soaking times reduce regeneration rates.

In garlic (*A. sativum* L.), soaking stem discs in a liquid B5 medium containing 0.5% colchicine led to the successful generation of tetraploid plants with thicker leaves, enlarged guard cells, and increased secondary metabolite concentrations (Dixit & Chaudhary, 2014). For *A. cepa* L. var. aggregatum G. Don, treatment with 250 µmol/L colchicine for four days yielded a variation rate of 42.22%, producing slower-growing tetraploid plants with thicker leaves and larger bulbs (Ren et al., 2018). In a study on the “Tawangmangu Baru” garlic variety from Indonesia, tetraploid plants were successfully induced by soaking stem disc explants in a 0.1% colchicine solution for 24 or 48 h (Hailu, Wiendi & Dinarti, 2020). Additionally, *A. wakegi* shoots cultured in a medium containing two g/L colchicine for 3 days also produced tetraploid plants with distinct morphology compared to diploid plants (Adaniya & Tamaki, 1991). This morphological divergence is largely attributable to the effect of colchicine on the mitotic spindle, which impedes normal chromosome segregation during mitosis and leads to the formation of tetraploid cells. When the nucleus contains four sets of chromosomes (4*n*), the increased DNA content results in larger cell size, which consequently causes observable morphological differences between tetraploid and diploid plants. Similar findings have been reported in other species. For instance, colchicine-induced tetraploid *Citrus limon* exhibited larger stomata and reduced stomatal density compared with diploid plants (Bhuvaneswari, Thirugnanasampandan & Gogulramnath, 2020), while tetraploid *Trachyspermum ammi* (Ajowan) showed increased stomatal size and decreased stomatal frequency (Sadat Noori et al., 2017). However, when colchicine concentration or exposure duration is excessive, the microtubule network is severely disrupted, causing irreversible spindle malfunction and mitotic arrest. This cytotoxic effect inhibits regeneration and can ultimately lead to cell death rather than successful chromosome doubling, explaining the sharp decline in survival observed at higher doses in this study.

Furthermore, the results showed that short-term inoculation treatment combined with 12 h of colchicine soaking had no significant effect on polyploid induction. In contrast, long-term inoculation treatment combined with 24 h of soaking significantly increased the variation rate, with 100% variation observed after a 10-day inoculation period. One tetraploid plant with larger stomata and increased ploidy was produced. The enhanced variation observed after longer pre-culture may be attributed to physiological activation and synchronization of the explant cells. Extended pre-culture likely reactivates cell division in meristematic and subepidermal layers, thereby increasing the proportion of cells entering the mitotic phase when exposed to colchicine. Since colchicine acts by inhibiting spindle formation during metaphase, a higher number of actively dividing cells increases the likelihood of successful chromosome doubling. These findings are consistent with previous reports that extended pre-treatment periods can enhance colchicine uptake or alter the cell cycle status to favor polyploid induction.

Similar phenomena have been reported in other bulbous species. For instance, in *Lilium regale*, culturing scales in a shoot induction medium for 10 days before soaking in a 0.01% colchicine solution for 24 h resulted in a 27.3% tetraploid induction rate (Jeloudar et al., 2019). Similarly, in *Lilium davidii* var. unicolor, pre-culturing scales for 15 days followed by soaking in a 0.025% colchicine solution for 24 h yielded the highest tetraploid induction rate (Li et al., 2020). In *L. longiflorum*, pre-culturing bulb explants for 10 days followed by cultivation in MS medium containing 20 mg/L colchicine for 2 days produced the highest number of tetraploid plants (Kafawin & Chen, 1991). These results suggest that pre-culturing can stimulate mitosis in explant cells, and since the shoot primordia originate from a single cell, treating the explants before shoot formation can induce polyploidy by doubling cells in the metaphase of mitosis, eventually forming tetraploid adventitious shoots (Li et al., 2020).

Although the 10-day pre-culture treatment in *A. grayi* produced a 100% variation rate and a 20% tetraploid induction rate, these results were based on a small sample size (five regenerated shoots). Therefore, the findings should be interpreted cautiously. Nevertheless, this observation provides valuable preliminary evidence that prolonged pre-culture enhances explant responsiveness to colchicine treatment, offering a promising direction for future breeding and optimization of polyploid induction protocols in *Allium* species.

Consistent with these observations, in native Matsu shallot, a 3-day pre-culture period resulted in the production of some shoots, while a 10-day pre-culture period produced more variant plants. It can be inferred that a 3-day pre-culture may not fully activate the scale explants, leading to the formation of some diploid plants. Therefore, a 10-day pre-culture followed by colchicine treatment is the optimal method for generating variation and tetraploid in native Matsu shallot.

However, mixoploids frequently appeared after colchicine treatment. This phenomenon may arise from the limited penetration of colchicine into specific tissues or the layered structure of the shoot apical meristem, which prevents uniform chromosomal doubling across all cell layers (Dhooghe et al., 2011; Sattler, Carvalho & Clarindo, 2016). To overcome this problem, repeated subculturing of shoot tips or nodal segments can facilitate the segregation of uniform tetraploid lines, as mitotic divisions may gradually eliminate diploid sectors. Alternatively, adventitious shoot regeneration from single-cell origins—such as callus induction or somatic embryogenesis—can also enhance the likelihood of obtaining homogeneous tetraploid plants (Gao et al., 2022).

These findings collectively emphasize the importance of optimizing pre-culture conditions to improve polyploid induction efficiency in *Allium* species. For breeding programs, this finding implies that refining pre-culture conditions, including the physiological status of explants, and culture duration, could enhance the production of stable tetraploid lines with desirable agronomic traits such as larger bulb size, improved stress tolerance, and modified secondary metabolite profiles.

From a research perspective, further studies should focus on elucidating the physiological and molecular mechanisms underlying colchicine uptake and cell cycle regulation during pre-culture. Comparative transcriptomic or proteomic analyses between diploid and induced polyploid tissues may reveal key pathways associated with chromosomal doubling. Moreover, integrating pre-culture optimization with alternative antimitotic agents or physical treatments (*e.g.*, oryzalin, trifluralin, or temperature shifts) could provide safer and more efficient methods for polyploid induction in Allium and related species.

## Conclusions

Native Matsu shallot, commonly consumed by local residents in Matsu, was successfully induced into tetraploid plants and verified by flow cytometry analysis in this study. The tetraploid plants were characterized by lower stomatal density and larger stomatal size compared to diploids. In this experiment, the effects of colchicine with different concentrations and treatment time on the induction of polyploid Matsu shallot (*Allium grayi* Regel) were studied *in vitro*. The results clearly indicated that as colchicine concentration and soaking time increased, the survival rate tended to decrease. In addition, a long-term pre-culture in shoot induction medium of the quartered bulbs prior to soaking in one g/L colchicine for 24 h significantly enhanced plant variation, with the 10-day pre-culture group showing the best results, achieving a 100% variation rate and a 20% tetraploid induction rate. These findings can be applied for future commercial production, selecting superior lines to replace the collection of wild populations, thus contributing to the program of breeding, conservation and sustainability of Matsu’s native shallot populations.

##  Supplemental Information

10.7717/peerj.20790/supp-1 Supplemental Information 1 Raw data The effects of all colchicine treatments on the explants, as well as the differences in stomatal characteristics between diploid and tetraploid plants.

## Additional Information and Declarations

### Data Availability

The following information was supplied regarding data availability: The flow cytometry data are available at Figshare: chen, ting syuan (2025). *Allium grayi* Regel polyploid. figshare. Dataset. https://doi.org/10.6084/m9.figshare.29554940.v2. The raw measurements are available in the Supplemental File.
